# A Colloidal Gold Immunochromatographic Strip Based on a Conserved Epitope Peptide for Rapid Detection of Antibodies Against Avian Infectious Bronchitis Virus

**DOI:** 10.3390/microorganisms14091887

**Published:** 2026-08-25

**Authors:** Ling Liu, Kang Zhao, Chang-Run Zhao, Tao-Ni Zhang, Yi Li, Qin Wu, Qi Wang, Chuan-Rui Yang, Wen-Qing Zhao, Qiu-Ying Chen, Tianchao Wei, Teng Huang, Jianni Huang, Meilan Mo

**Affiliations:** 1 College of Animal Science and Technology, Guangxi University, Nanning 530004, China; liuling5764@163.com (L.L.); zhaokang0519@163.com (K.Z.); zcrun628@163.com (C.-R.Z.); zhangtaoni66@163.com (T.-N.Z.); lllyww1011@163.com (Y.L.); woo19965459282@163.com (Q.W.); wangqi20021028@163.com (Q.W.); 17685931850@163.com (C.-R.Y.); m18178134190@163.com (W.-Q.Z.); gxdxchenqy@126.com (Q.-Y.C.); tcwei88@126.com (T.W.); tenhhwang@163.com (T.H.); jiannihuang@gxu.edu.cn (J.H.); 2 Guangxi Zhuang Autonomous Region Engineering Research Center of Veterinary Biologics, Nanning 530004, China; 3 Guangxi Key Laboratory of Animal Reproduction, Breeding and Disease Control, Nanning 530004, China

**Keywords:** infectious bronchitis virus, epitope peptide, colloidal gold, immunochromatographic strip, rapid detection

## Abstract

Avian infectious bronchitis virus (IBV) is widely distributed worldwide and causes substantial economic losses to the poultry industry. Because IBV undergoes frequent mutation, prevention and control of infection remain challenging. Immunization is an important measure for the prevention and control of IB. Therefore, there is an urgent need for a rapid, sensitive, specific, and convenient method for the detection of antibodies against IBV. In this study, we firstly developed an indirect colloidal gold immunochromatographic strip for the rapid detection of antibodies against IBV based on a conserved epitope peptide. The recombinant epitope peptide recognized by N2D5 monoclonal antibody (mAb) against the N protein of IBV was expressed as a GST fusion protein (GST-N2D5) based on the conserved antigenic epitope previously identified in our laboratory. Colloidal gold-labeled GST-N2D5 was used as the detection reagent to generate visual signals. Rabbit anti-chicken IgY and mouse anti-GST mAb were immobilized on the nitrocellulose membrane as the test line (T line) and control line (C line), respectively. The optimal pH and optimal protein concentration for conjugation of gold nanoparticles (AuNPs) with GST-N2D5 were pH 8.5 and 72 µg/mL, respectively. Specificity was evaluated using common avian pathogens, and no cross-reactivity was observed. The detection limit of the strip for IBV-positive serum was 1:180. In addition, the assay showed good reproducibility and stability, and results could be observed within 5 min without any specialized equipment. Clinical chicken serum samples were tested using both the developed strip and an enzyme-linked immunosorbent assay (ELISA), and the strip showed high agreement with the ELISA. In conclusion, the established immunochromatographic strip is rapid, sensitive, specific, and easy to operate, and therefore has potential as an on-site tool for the rapid detection of antibodies against IBV, particularly in resource-limited settings.

## 1. Introduction

Avian infectious bronchitis (IB) is an acute and highly contagious disease of poultry caused by avian infectious bronchitis virus (IBV) [1]. IB mainly affects the respiratory, reproductive, and urinary systems of poultry [2], and clinically manifests as sneezing, tracheal rales, and dyspnea, leading to poor growth performance, reduced egg production, and impaired egg quality [3]. Owing to the widespread prevalence and frequent genetic variation of IBV, numerous serotypes and genotypes have emerged, with limited or no cross-protection among them [4]. This extensive genetic diversity greatly complicates the diagnosis, surveillance, and control of IB. Vaccination is the main measure for the prevention and control of IB. Antibody detection can provide scientific basis for the assessment of immune efficacy, disease monitoring and control. Therefore, the development of rapid, specific and simple antibody detection methods has significant practical significance.

Among the four structural proteins of IBV, namely the spike (S), nucleocapsid (N), envelope (E), and membrane (M) proteins [5], the selection of an appropriate detection target is critical for diagnostic assay development. The S protein is the major glycosylated protein responsible for inducing neutralizing antibodies but is prone to mutation [6]. The E protein is a low-abundance structural protein involved mainly in viral assembly and budding. The M protein is the most abundant envelope protein; however, it is not very conserved and is highly glycosylated, which interferes with detection [7,8]. In contrast, the N protein is more highly conserved among different IBV strains, showing a sequence identity of 91.0–96.5% [9]. Moreover, the N protein is abundantly expressed during infection and can induce high levels of cross-reactive antibodies as well as robust T-cell responses, making it a promising target for diagnostic applications [10,11]. Previous studies have demonstrated the applicability of the N protein in serological assays. For example, Zhang et al. (2023) developed a sensitive indirect ELISA based on the recombinant N160 protein for detecting IBV antibodies [12]. In addition, Finger et al. (2018) reported that an ELISA combined with Western blot for antibodies against the IBV N protein achieved a sensitivity of 90.34% and a specificity of 90.16% [13]. These findings support the N protein as an important antigen for IBV antibody detection.

Specific antigenic epitopes have also been widely used in the development of antibody detection assays. Antigenic epitopes can elicit humoral and specific cellular immune responses and play important roles in antiviral immunity [14]. Epitope-based methods have been successfully applied for the detection of antibodies against Dengue virus and GB virus C with high sensitivity and specificity [15,16]. Ding et al. (2015) developed multiple peptide antigens derived from the S protein of IBV for use in a highly sensitive and specific ELISA for detecting IBV-specific antibodies in chicken serum samples [17]. However, there is no antibody detection method based on the epitope peptide of the conserved N protein. Our previous study identified a B-cell epitope with the amino acid sequence IPLNRGRGGRST on the N protein of the GX-YL5 strain, which can be specifically recognized by the monoclonal antibody N2D5. Notably, this epitope reacted with monospecific sera against all seven IBV serotypes prevalent in Guangxi, China, indicating that it is highly conserved and broadly reactive [18]. These characteristics suggest that this epitope is a promising antigenic candidate for the development of antibody detection methods for IBV.

At present, the main methods for detecting IBV antibodies include an ELISA, Western blot, and an indirect fluorescent antibody assay (IFA). However, these methods are labor-intensive, time-consuming, and costly, and they generally require specialized instruments and trained personnel, which limits their use in field settings. By contrast, colloidal gold immunochromatographic technology offers several advantages, including simplicity, rapidity, low cost, and minimal equipment dependence, and is therefore well suited for on-site testing [19,20,21].

So far, there have been a few reports on colloidal gold immunochromatographic detection methods related to IBV [22,23]. However, Zhao et al. (2024) developed an assay for the detection of IBV antigens instead of antibodies [22]. Although Zhang et al. established an indirect gold immunochromatographic assay for IBV antibody detection, this assay was based on the recombinant N protein rather than an epitope peptide [23]. Currently, rapid antibody detection methods based on broadly reactive conserved epitopes remain limited. Accordingly, in the present study, we developed a rapid, sensitive, specific, and easy-to-operate immunochromatographic strip for the detection of IBV antibodies based on a broadly reactive epitope derived from the N protein of the GX-YL5 strain [18], aiming to provide a practical tool for field diagnosis, immune monitoring, and epidemiological surveillance. As far as we know, this is currently the first colloidal gold immunochromatographic strip method for detecting IBV antibodies based on the conserved epitope peptide of the N protein.

## 2. Materials and Methods

### 2.1. Materials and Reagents

The positive sera against infectious bronchitis virus (IBV), avian influenza virus (AIV), Newcastle disease virus (NDV), infectious laryngotracheitis virus (ILTV), avian metapneumovirus (aMPV), infectious bursal disease virus (IBDV), avian leukosis virus (ALV), Marek’s disease virus (MDV), avian reovirus (ARV), *Mycoplasma*, *Escherichia coli*, *Salmonella* and *Chlamydia psittaci* were collected and maintained in our laboratory. The poultry serum samples were archived samples obtained from routine animal disease surveillance on commercial farms and were not collected specifically for this study. Rabbit anti-chicken IgY and bovine serum albumin (BSA) were purchased from Solarbio, Beijing, China. Anti-GST mouse mAb was purchased from TransGen Biotech, Beijing, China. Tetrachloroauric acid (HAuCl4) was purchased from Sigma-Aldrich, St. Louis, MO, USA. Sodium citrate, sucrose, Tween-20, potassium carbonate (K_2_CO_3_), and Tris(hydroxymethyl)aminomethane (Tris) were purchased from Sinopharm Chemical Reagent Co., Ltd., Shanghai, China. The recombinant pGEX-6P-GST-N2D5 expression vector was prepared and stored in our laboratory. The colloidal gold solution, glass fiber membrane, polyvinylchloride (PVC) baseboard, bibulous paper and nitrocellulose membrane (NC membrane) were purchased from Shanghai JY Biotechnology Co., Ltd., Shanghai, China. All chemicals were of analytical grade.

### 2.2. Expression and Purification of GST-N2D5 Protein

The recombinant pGEX-6P-GST-N2D5 expression vector was used to transform *E. coli* BL21 (DE3) Star competent cells. The resulting recombinant clones were cultured in 100 mL LB broth supplemented with 100 µg/mL ampicillin at 37 °C for 6 h with shaking at 250 rpm. Expression of the recombinant N2D5 protein was induced by adding isopropyl-β-D-thiogalactopyranoside (IPTG) to a final concentration of 0.1 mM, followed by incubation at 37 °C for 5 h with shaking at 250 rpm. The cells were harvested by centrifugation at 4000 rpm for 10 min at 4 °C, and the resulting pellet was resuspended in lysis buffer (6.3% glycerol, 0.58% NaCl, 0.6% Tris, 0.0015% EDTA, 0.0007% DTT, supplemented with 0.1 mM PMSF). The resuspended cells were subjected to seven sonication cycles (10 s each, 70 Hz) and then centrifuged again. The supernatant was purified using a GST Fusion Protein Purification Kit (GenScript, Nanjing, China). The protein concentration was determined using the Qubit™ Protein Assay Kit (Thermo Fisher Scientific, Waltham, MA, USA) according to the manufacturer’s instructions. The expressed GST-N2D5 protein was identified by SDS-PAGE and Western blot analysis.

### 2.3. Synthesis and Optimization of AuNPs

The preparation of AuNPs was performed according to Chen et al. (2023) with minor modifications [24]. Initially, the temperature was set at 137 °C, and 140 mL of ultrapure water was heated to boiling in an oil bath. To obtain AuNPs with an average diameter of 40 nm, a series of AuNPs of different sizes was prepared by adding 5, 10, 15, or 20 mL of 1% sodium citrate (SC). When the temperature reached 137 °C, 1 mL of 1% tetrachloroauric acid solution was rapidly added. After 1 min, Tris solution (5 mL, 0.1 M) was rapidly added to the mixture. After 30 min of reaction, the temperature was adjusted to 100 °C. Once the temperature reached 100 °C, 1 mL of 1% tetrachloroauric acid was rapidly added to the mixture again. The previous addition step was repeated, and the resulting colloidal gold was stored at 4 °C protected from light.

The UV-Vis spectra of the AuNPs were acquired using a UV-Vis spectrophotometer (Shimadzu, Kyoto, Japan). The particle size of the AuNPs was determined using a particle size analyzer (Malvern Panalytical, Malvern, UK). AuNPs with the narrowest UV-Vis absorption peak and a particle size of 40 nm were selected for transmission electron microscopy (TEM) characterization and subsequent experiments.

### 2.4. Preparation of the AuNPs-GST-N2D5 Protein Conjugate

#### 2.4.1. Optimization of the Labeling pH

The method for optimizing the pH was based on a previously reported procedure [25]. To optimize the pH for protein labeling of the colloidal gold solution, 1.0 mL of colloidal gold solution was adjusted with 0, 10, 20, 30, 35, 45, or 50 µL of 0.1 M K_2_CO_3_. After vortex mixing, 30 µL of GST-N2D5 protein was added and the mixture was incubated at room temperature for 30 min. Then, 100 µL of 10% BSA was added for blocking for 1 h. The labeled complexes were collected by centrifugation at 10,000 r/min and 4 °C for 40 min, and the pellets were resuspended in 100 µL of 50 mM Tris-HCl buffer (pH 8.5) containing 10% sucrose, 5% BSA, and 1% Tween-20. The resuspended conjugates were dispensed onto the conjugate pads, dried at 37 °C for 2.5 h, sealed, and stored under dry conditions at 4 °C. The immunochromatographic strips prepared with conjugate pads obtained at different pH values were used to detect IBV-positive serum, with SPF chicken serum as the negative control. Serum samples were diluted 1:50 in 50 mM Tris-HCl buffer (pH 8.5) containing 2% Tween-20 before testing. The optimal pH was determined based on the clearest T line observed for the positive serum sample.

#### 2.4.2. Optimization of the GST-N2D5 Labeling Concentration

Optimization of the protein labeling concentration was performed using colloidal gold solution pre-adjusted to the optimal pH. Specifically, 1.0 mL of colloidal gold solution was adjusted to pH 8.5 with 35 µL of 0.1 M K_2_CO_3_, followed by the addition of the GST-N2D5 protein to final concentrations of 15, 30, 45, 60, 75, and 90 µg/mL. After vortex mixing, the mixtures were incubated at room temperature for 30 min, and then 100 µL of 10% BSA was added for blocking for 1 h. The labeled complexes were collected by centrifugation at 10,000 r/min at 4 °C for 40 min, and the pellets were resuspended in 100 µL of 50 mM Tris-HCl buffer (pH 8.5) containing 10% sucrose, 5% BSA, and 1% Tween-20. The resuspended conjugates were dispensed onto the conjugate pads, dried at 37 °C for 2.5 h, sealed, and stored under dry conditions at 4 °C. The strips prepared under different conditions were further compared using IBV-positive serum and a negative control. The minimum labeling concentration that produced the clearest T line for the positive sample was increased by 20% and defined as the optimal labeling concentration.

#### 2.4.3. Optimization of the Resuspension Ratio

The resuspension volume of the colloidal gold conjugate was optimized under the previously determined optimal labeling conditions. A total of 1.0 mL of colloidal gold solution was adjusted to pH 8.5 with 35 µL of 0.1 M K_2_CO_3_, and GST-N2D5 protein was added to a final concentration of 72 µg/mL. After incubation at room temperature for 30 min and blocking with 100 µL of 10% BSA for 1 h, the labeled complexes were collected by centrifugation at 10,000 r/min and 4 °C for 40 min. The pellets were resuspended in 50, 75, 100, 125, 150, or 200 µL of 50 mM Tris-HCl buffer (pH 8.5) containing 10% sucrose, 5% BSA, and 1% Tween-20, dispensed onto the conjugate pads, dried at 37 °C for 2.5 h, sealed, and stored under dry conditions at 4 °C. The strips prepared with different resuspension volumes were evaluated using IBV-positive serum and the negative control serum, and the optimal resuspension volume was determined based on the clearest T line for the positive serum sample.

### 2.5. Preparation of the Immunochromatographic Strip

The AuNPs-GST-N2D5 conjugate was applied to the conjugate pad and allowed to dry at 37 °C for a period of 2 h. The rabbit anti-chicken IgY (0.75 mg/mL) and anti-GST mouse mAb (0.5 mg/mL) were immobilized on the NC membrane using the XYZ large platform 3D induction scribing (Shanghai Gold Standard Biotechnology Co., Ltd., Shanghai, China) as the T line and C line, respectively. Following a drying period of 2.5 h at 37 °C, the sample pad, conjugate pad, NC membrane and absorbent paper were affixed to the PVC backing pad in a consecutive manner. They were then cut into strips measuring 4 mm in width for subsequent experimentation.

### 2.6. Determination of the Optimal Detection Time

The optimal detection time was determined based on changes in the colloidal gold signal intensity of the T line after detection of positive serum. A total of 100 µL of IBV antibody-positive serum diluted 50-fold with sample dilution buffer was added to the sample pad. The T line signal was recorded every 2 min using a colloidal gold strip reader. The time point at which the T line signal became stable was defined as the optimal detection time.

### 2.7. Principle and Result Readout of the Immunochromatographic Strip

**Figure 1:**
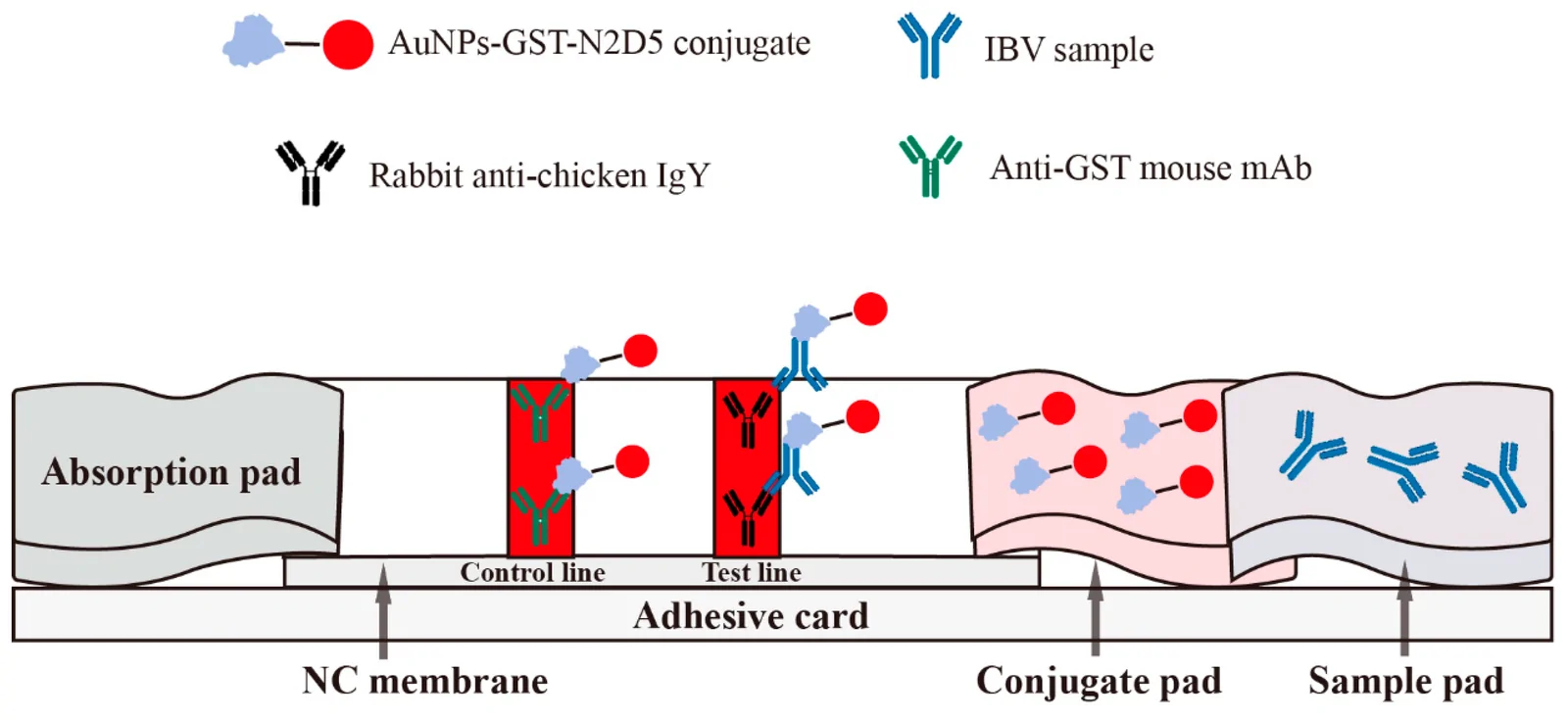
A schematic illustration of the working principle of the immunochromatographic strip for the IBV antibody.

As illustrated in Figure 1, the immunochromatographic strip consists of a sample pad with filtering function, a conjugate pad containing the AuNPs-GST-N2D5 conjugate, a NC membrane coated with rabbit anti-chicken IgY at the T line and mouse anti-GST mAb at the C line, an absorbent pad, and a backing card. After sample application, the liquid migrates sequentially from the sample pad through the conjugate pad and NC membrane to the absorbent pad by capillary action. During this process, antibodies in the serum bind to the AuNPs-GST-N2D5 conjugate, and the resulting immune complexes are captured by rabbit anti-chicken IgY at the T line, producing a visible red band. Meanwhile, excess AuNPs-GST-N2D5 conjugate is captured by the immobilized mouse anti-GST mAb at the C line, also generating a red band. In the absence of IBV-specific antibodies, only the C line appears red. In contrast, when IBV-specific antibodies are present, red bands appear at both the T and C lines.

### 2.8. Specificity Test of the Immunochromatographic Strip

The specificity of the immunochromatographic strip was evaluated using positive sera against common poultry pathogens, including AIV, NDV, ILTV, aMPV, IBDV, ALV, MDV, ARV, *Mycoplasma*, *E. coli*, *Salmonella*, and *C. psittaci*. SPF chicken serum was used as the negative control.

### 2.9. Sensitivity Testing of the Immunochromatographic Strip

To evaluate the sensitivity of the immunochromatographic strip, IBV-positive serum was serially diluted to 1:40, 1:60, 1:80, 1:100, 1:120, 1:140, 1:160, 1:180, and 1:200. Each diluted serum sample (100 µL) was applied to the strip, and SPF chicken serum was used as the negative control.

### 2.10. Repeatability Testing of the Immunochromatographic Strip

Intra-assay and inter-assay repeatability were assessed using IBV-positive serum and the negative control serum. For intra-assay repeatability, six strips were randomly selected from a single batch and used for detection, with each assay performed in triplicate. For inter-assay repeatability, two strips were randomly selected from each of three different batches and used to detect the same serum samples.

### 2.11. Stability Testing of the Immunochromatographic Strip

The stability and shelf life of the immunochromatographic strip were evaluated under different storage conditions. The assembled strips were individually packaged in dry, sealed bags and stored in the dark at 37 °C, 25 °C, and 4 °C. At monthly intervals, four strips were randomly selected from each storage condition and tested with IBV-positive serum diluted 1:50 and negative serum, with each assay performed in duplicate. Stability was assessed by continuously monitoring and comparing the test results obtained at different storage time points, and the shelf life of the strip was determined accordingly.

### 2.12. Detection of Clinical Samples

A total of 200 clinical chicken serum samples, collected from poultry farms in Guangxi, China, in 2023, were used to evaluate the performance of the immunochromatographic strips, and the results were compared with those obtained using a commercial IBV antibody ELISA kit (IDEXX Laboratories, Westbrook, ME, USA).

## 3. Results

### 3.1. Expression and Purification of GST-N2D5 Protein

**Figure 2:**
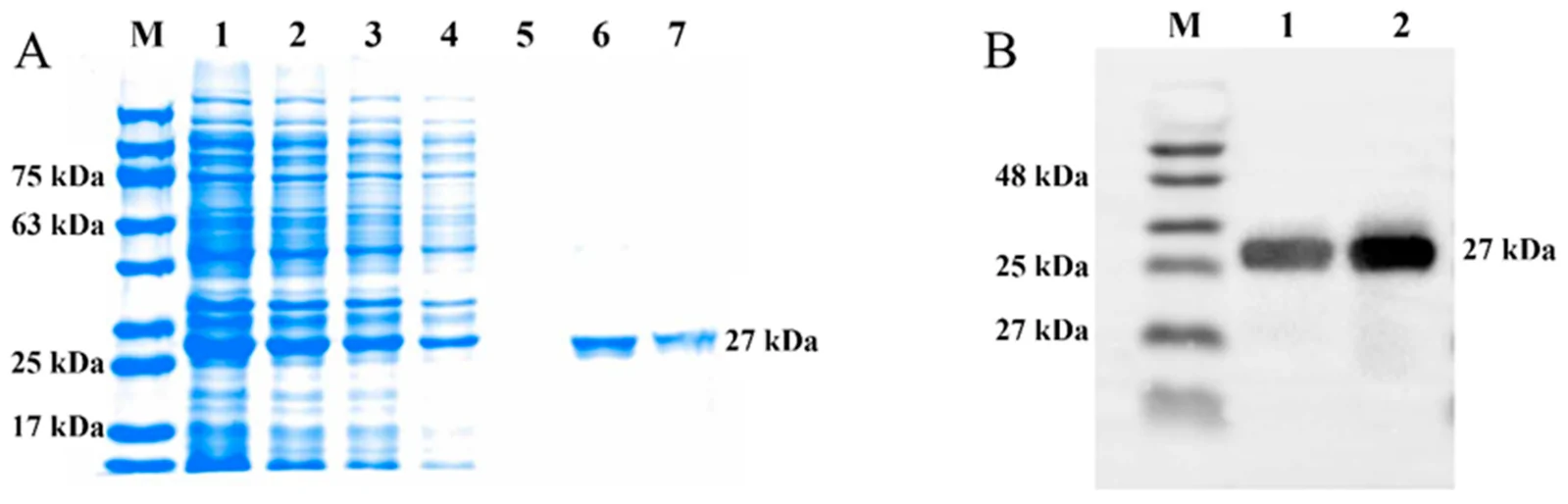
Identification of the GST-N2D5 protein by SDS-PAGE (**A**) and Western blot (**B**). (**A**) M: Protein Marker (10–180 kDa); 1–2: Flow through fluid; 3–5: Washing; 6–7: Elution. (**B**) M: Protein Marker (10–180 kDa); 1–2: IBV-positive serum.

The result showed that the GST-N2D5 protein was successfully expressed in soluble form. After purification, the protein concentration reached 2.5 mg/mL. SDS-PAGE analysis showed a single distinct band at approximately 27 kDa, which was in perfect agreement with the expected size (Figure 2A). Western blot analysis also showed a band at the same position, further confirming that it corresponded to the target protein (Figure 2B).

### 3.2. Characterization and Optimization of AuNPs

**Figure 3:**
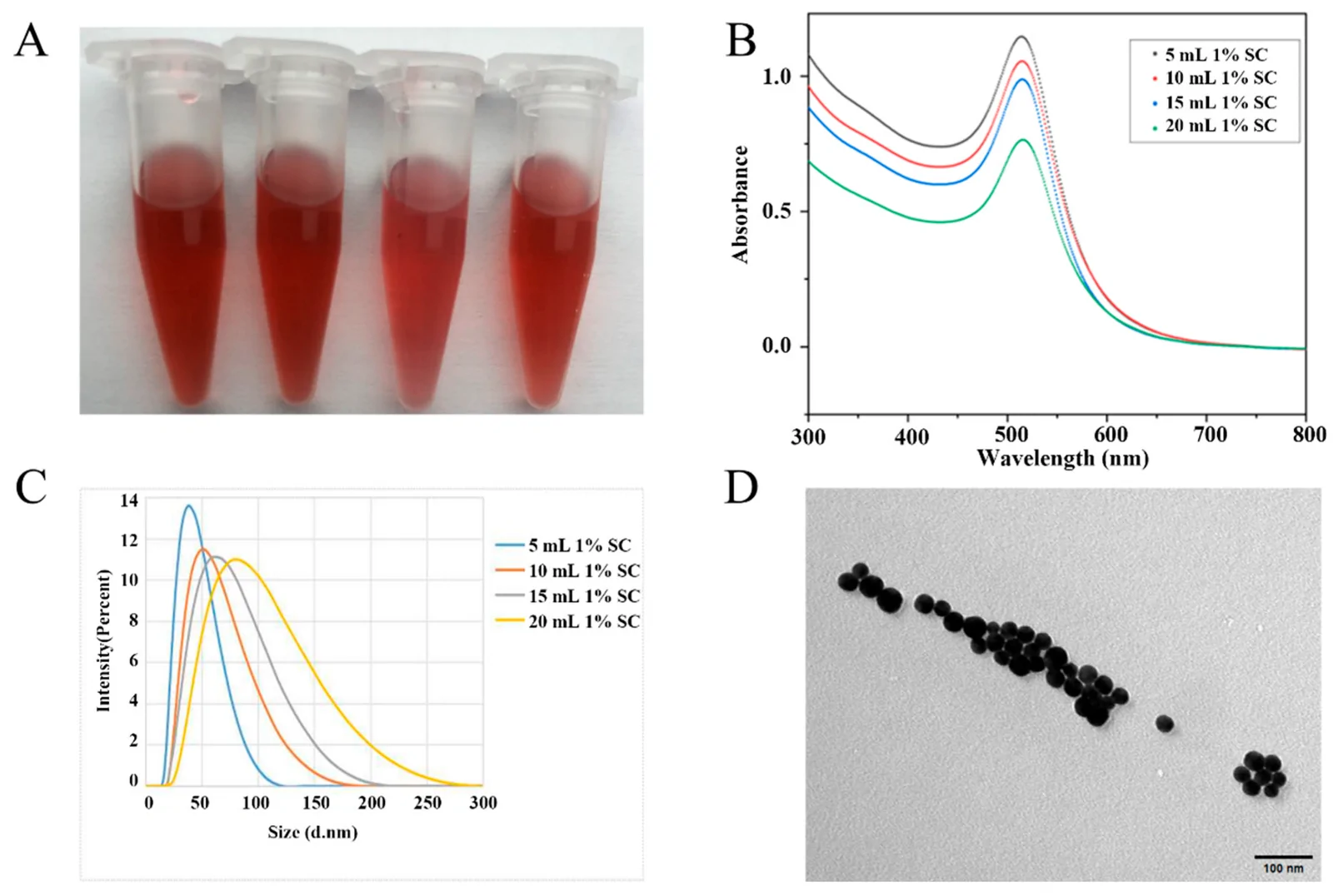
Colloidal gold characterization results prepared by 1% SC in different volumes. (**A**) Optical images (1: 5 mL 1% SC; 2: 10 mL 1% SC; 3: 15 mL 1% SC; 4: 20 mL 1% SC), (**B**) UV-vis spectra, (**C**) particle size chart and (**D**) TEM image.

By optimizing the volume of 1% SC, AuNPs with an average diameter of approximately 40 nm were prepared for use in the immunochromatographic strip. The optical images, UV-Vis spectra, particle size distributions, and TEM images of AuNPs prepared with different volumes of 1% SC were analyzed. The results showed that the AuNP solutions prepared with different volumes of 1% SC were all cherry red in color, with no obvious difference among groups, and no visible precipitation was observed (Figure 3A). The UV-Vis spectra showed a characteristic absorption peak at approximately 520 nm in all groups (Figure 3B). Among them, the AuNPs prepared with 5 mL of 1% SC showed the highest absorbance. The particle size analysis showed that the average diameter of the AuNPs prepared with 5 mL of 1% SC was close to 40 nm, and the particle size increased with increasing volume of the reducing agent (Figure 3C). To further determine the size and morphology of the AuNPs prepared with 5 mL of 1% SC, TEM analysis was performed. The results showed that these AuNPs were nearly spherical in shape and uniformly distributed, with an average diameter of approximately 40 nm (Figure 3D). Therefore, the AuNPs prepared with 5 mL of 1% SC were selected for subsequent experiments.

### 3.3. Preparation of the AuNPs-GST-N2D5 Protein Conjugate

#### 3.3.1. Optimization of the Labeling pH

**Figure 4:**
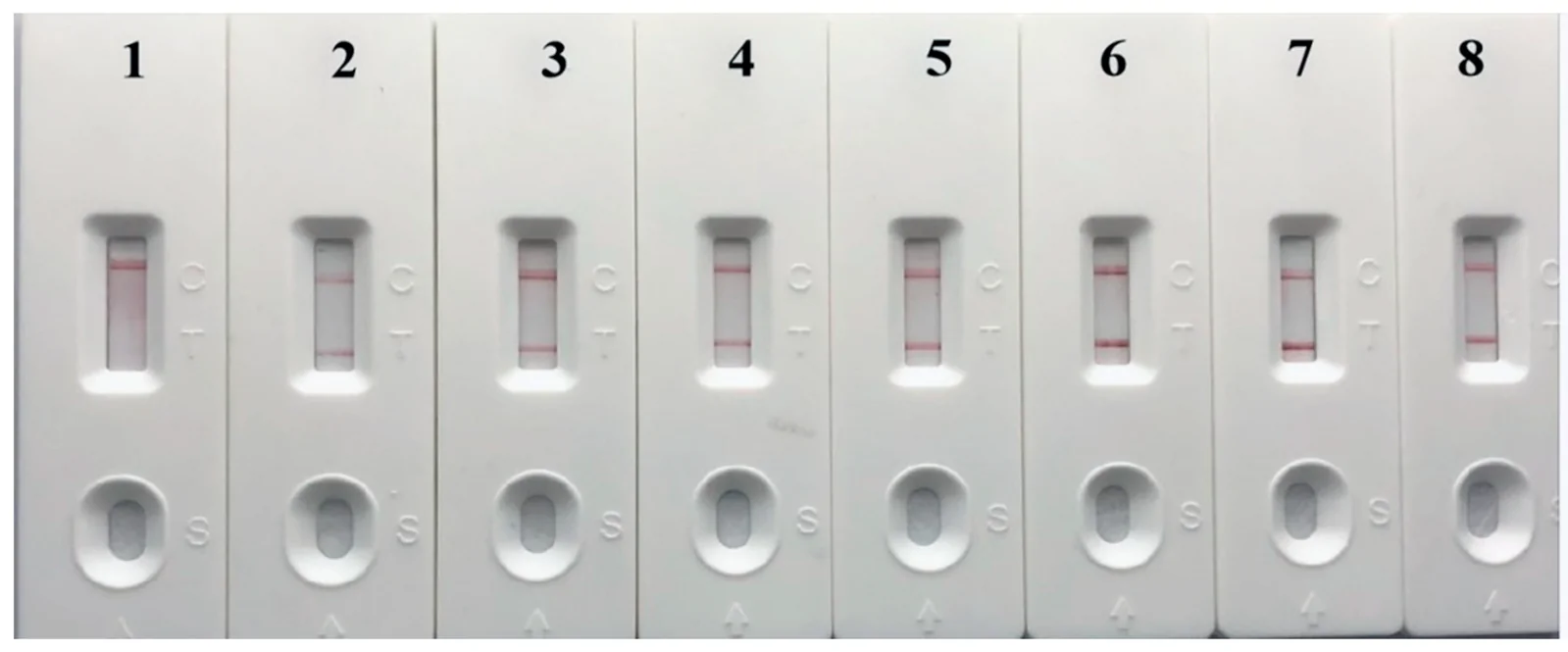
Optimization of the labeling pH for the immunochromatographic strip. 1: Negative control; 2: 0 µL K_2_CO_3_ (0.1 M); 3: 10 µL K_2_CO_3_ (0.1 M); 4: 20 µL K_2_CO_3_ (0.1 M); 5: 30 µL K_2_CO_3_ (0.1 M); 6: 35 µL K_2_CO_3_ (0.1 M); 7: 45 µL K_2_CO_3_ (0.1 M); 8: 50 µL K_2_CO_3_ (0.1 M).

To determine the optimal pH for GST-N2D5 labeling, the pH of 1 mL of colloidal gold solution was adjusted by adding different volumes of 0.1 M K_2_CO_3_. As shown in Figure 4, with increasing volume of 0.1 M K_2_CO_3_, the color intensity of both lines gradually increased. The clearest C line and T line were observed when 35 µL of 0.1 M K_2_CO_3_ was added, corresponding to pH 8.5. When the added volume exceeded 35 µL, the color intensity of both lines gradually decreased. Therefore, the optimal pH for labeling GST-N2D5 with 1 mL of colloidal gold solution was determined to be 8.5, and this condition was used in subsequent experiments.

#### 3.3.2. Optimization of the GST-N2D5 Labeling Concentration

**Figure 5:**
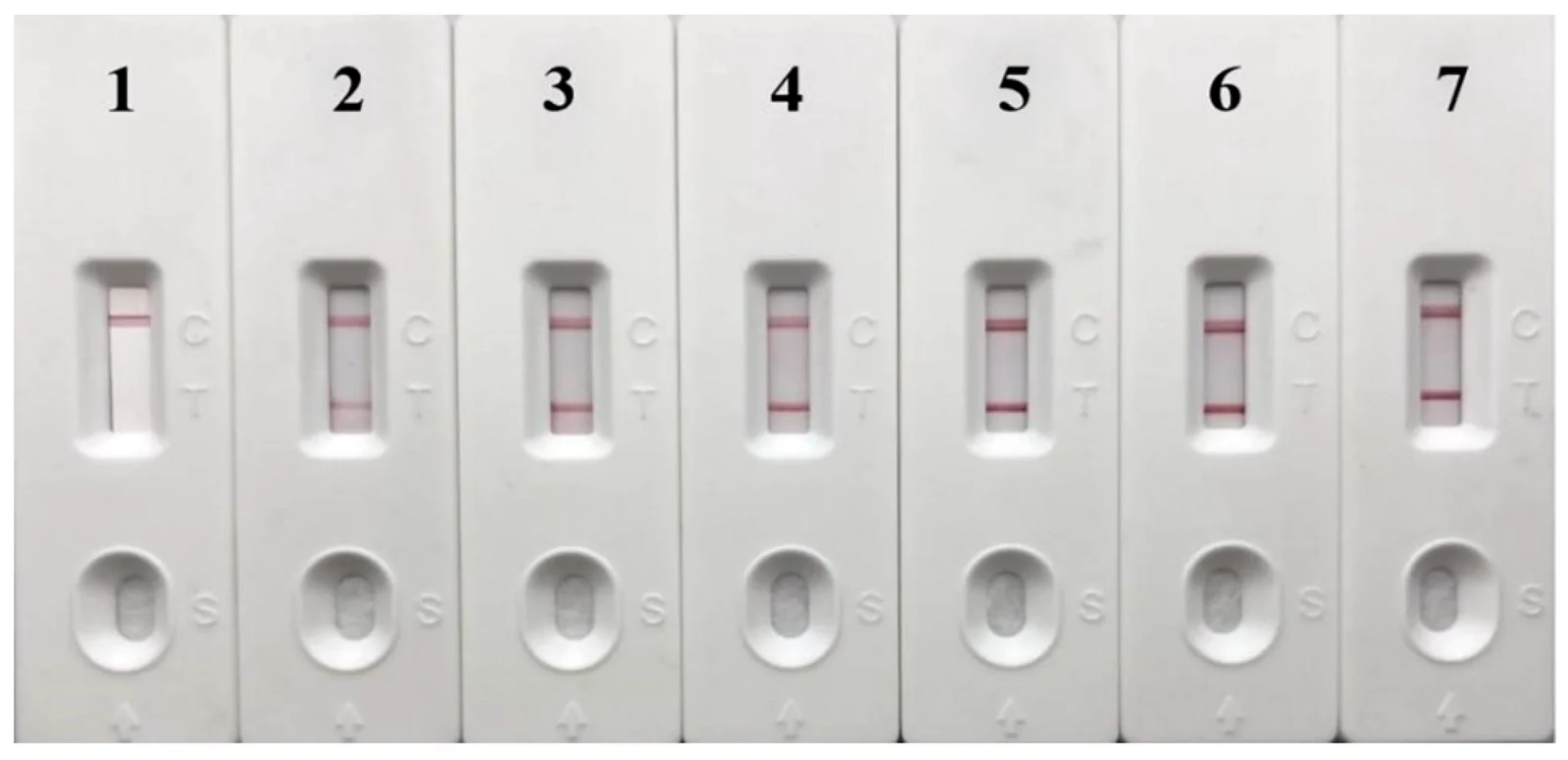
Optimization of the labeling concentration for the immunochromatographic strip. 1: Negative control; 2: 15 µg/mL; 3: 30 µg/mL; 4: 45 µg/mL; 5: 60 µg/mL; 6: 75 µg/mL; 7: 90 µg/mL.

Different concentrations of GST-N2D5 were added to 1 mL of colloidal gold solution to determine the optimal labeling concentration. As shown in Figure 5, the color intensity of the C line and T line increased gradually with increasing GST-N2D5 concentration. When the GST-N2D5 concentration reached 60 µg/mL, the strongest color intensity of both lines was observed and the membrane background remained clear. Further increases in GST-N2D5 concentration did not further enhance the color intensity of either line. Therefore, the minimum GST-N2D5 concentration required for labeling was determined to be 60 µg/mL. To provide a practical safety margin and improve the labeling efficiency, stability, and reproducibility of the AuNP–GST-N2D5 conjugate, this concentration was increased by 20% and used as the optimal labeling concentration. Thus, the optimal labeling concentration was set at 72 µg/mL and used in subsequent experiments.

#### 3.3.3. Optimization of the Resuspension Ratio

**Figure 6:**
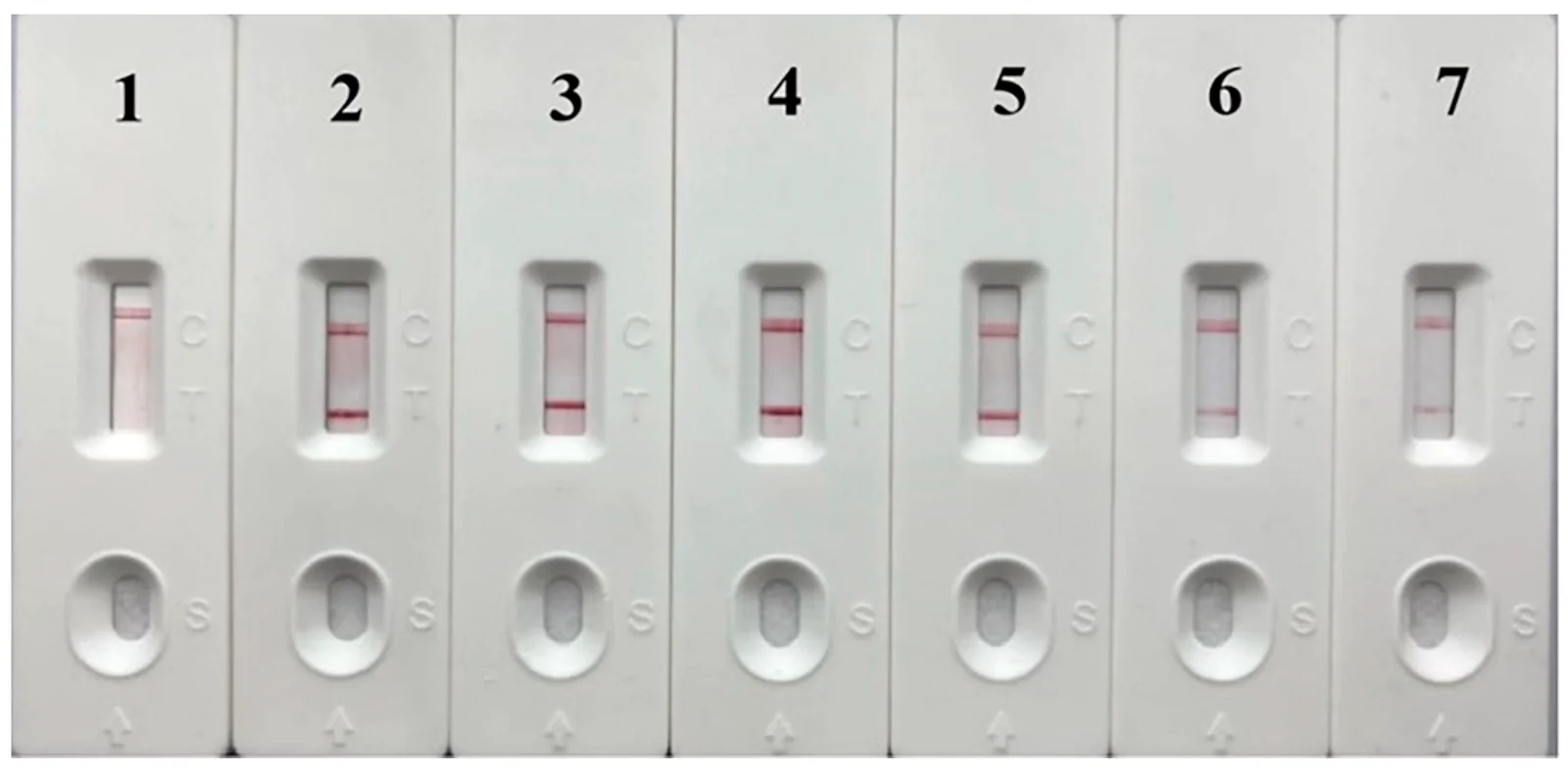
Optimization of the resuspension ratio for the immunochromatographic strip. 1: Negative control; 2: 50 µL complex solution; 3: 75 µL complex solution; 4: 100 µL complex solution; 5: 125 µL complex solution; 6: 150 µL complex solution; 7: 200 µL complex solution.

Different resuspension volumes of the colloidal gold conjugate were evaluated under the previously determined optimal pH and GST-N2D5 labeling concentration. As shown in Figure 6, when the resuspension volume was 100 µL, a clearer C line and T line were observed on the strip for the positive serum sample. Therefore, the optimal resuspension volume of the colloidal gold conjugate was determined to be 100 µL for each 1 mL of the colloidal gold labeling system.

### 3.4. Optimal Detection Time

**Figure 7:**
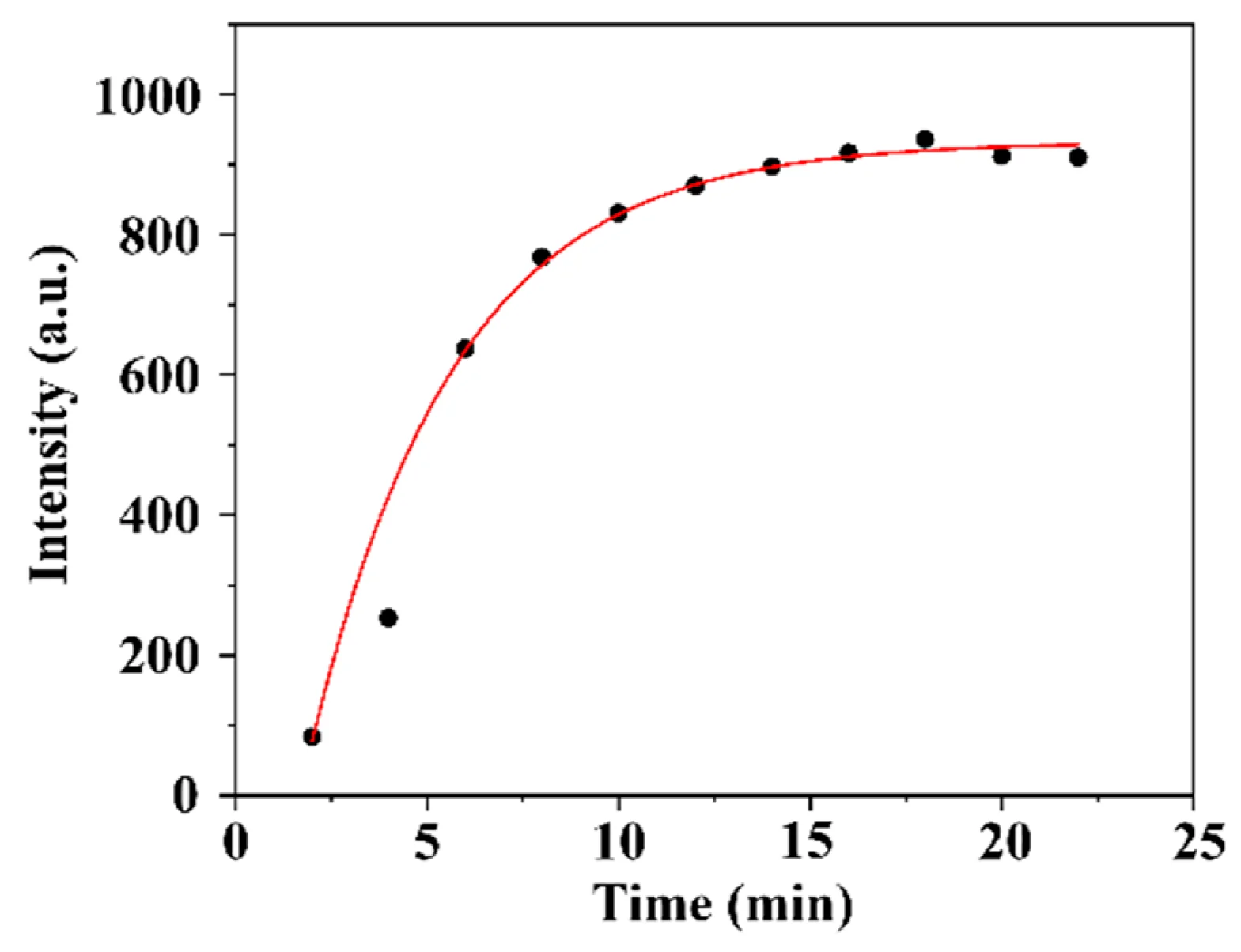
Optimization of the detection time for the immunochromatographic strip.

The optimal detection time of the immunochromatographic strip was determined using a colloidal gold strip reader by measuring the colloidal gold signal intensity at the T line every 2 min after sample loading. As shown in Figure 7, the signal intensity increased steadily over time, and the test result could be preliminarily observed within 5 min. The signal gradually reached a stable level at approximately 16 min. These results indicated that 16 min was the recommended detection time for the strip, particularly for samples with low IBV antibody titers.

### 3.5. Specificity of Immunochromatographic Strip

**Figure 8:**
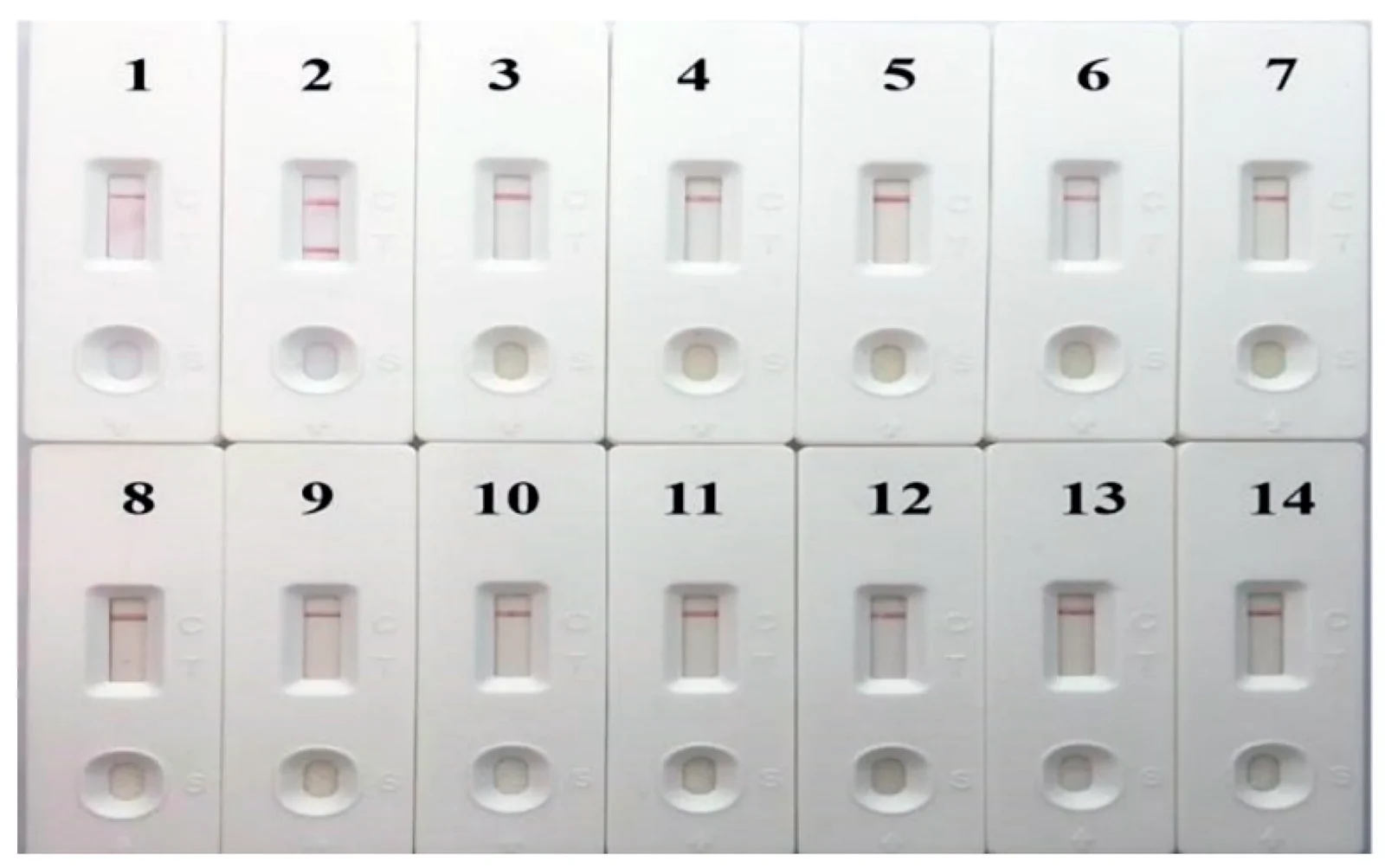
The specificity of the immunochromatographic strip. 1: Negative control; 2: IBV-positive serum; 3: AIV-positive serum; 4: NDV-positive serum; 5: ILTV-positive serum; 6: aMPV-positive serum; 7: IBDV-positive serum; 8: ALV-positive serum; 9: MDV-positive serum; 10: MS-positive serum; 11: *Salmonella*-positive serum; 12: *E. coli*-positive serum; 13: ARV-positive serum; 14: *C. psittaci*-positive serum.

The specificity of the immunochromatographic strip was assessed using positive sera against common poultry pathogens. After 5-fold dilution, all strips showed a clear C line, confirming the validity of the assay. A clear T line was observed only for the IBV-positive serum, whereas no T line was detected for the other positive sera or the negative control serum (Figure 8). These results indicated that the strip showed no cross-reactivity with the tested pathogens and had high specificity for the detection of IBV antibodies.

### 3.6. Sensitivity of Immunochromatographic Strip

**Figure 9:**
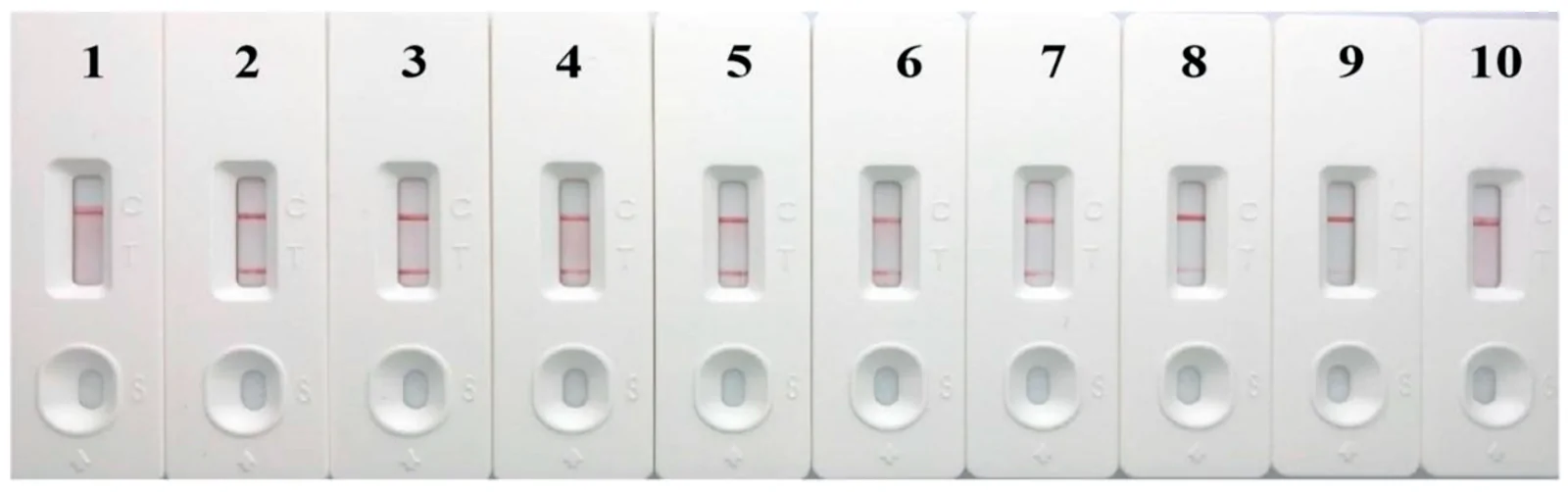
Sensitivity of immunochromatographic strip. 1: Negative control; 2: 1:40; 3: 1:60; 4: 1:80; 5: 1:100; 6: 1:120; 7: 1:140; 8: 1:160; 9: 1:180; 10: 1:200.

Serial dilutions of IBV-positive serum were used to evaluate the sensitivity of the immunochromatographic strip. As shown in Figure 9, the color intensity of the T line gradually decreased with increasing dilution of the IBV-positive serum. A weak color signal was still visible at a dilution of 1:180, indicating that the limit of detection of the immunochromatographic strip was 1:180.

### 3.7. Repeatability of Immunochromatographic Strip

**Figure 10:**
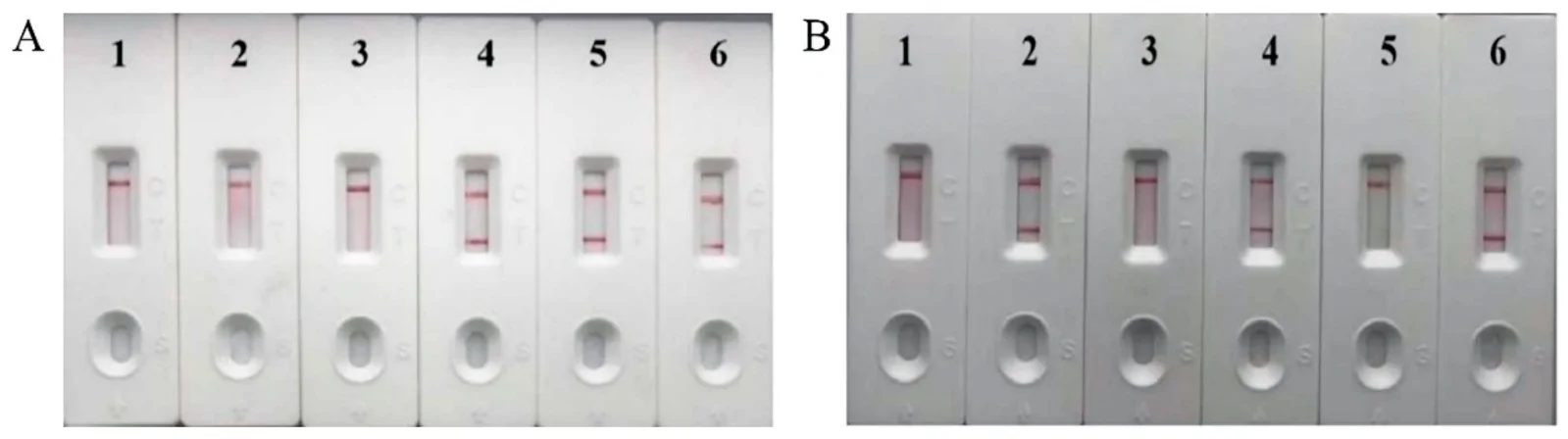
Repeatability of immunochromatographic strip. (**A**) Intra-assay repeatability 1–3: Negative control; 4–6: IBV-positive serum. (**B**) Inter-assay repeatability. 1, 3, 5: Negative control; 2, 4, 6: IBV-positive serum.

The repeatability of the immunochromatographic strip was assessed using IBV-positive serum, including intra-assay testing within a single batch and inter-assay testing across different batches. As shown in Figure 10, no obvious difference was observed among the immunochromatographic strips randomly selected from the same batch or from different batches when tested with sera containing the same concentration of IBV. These results indicate that the strip has good intra-assay and inter-assay reproducibility.

### 3.8. Stability of Immunochromatographic Strips

**Figure 11:**
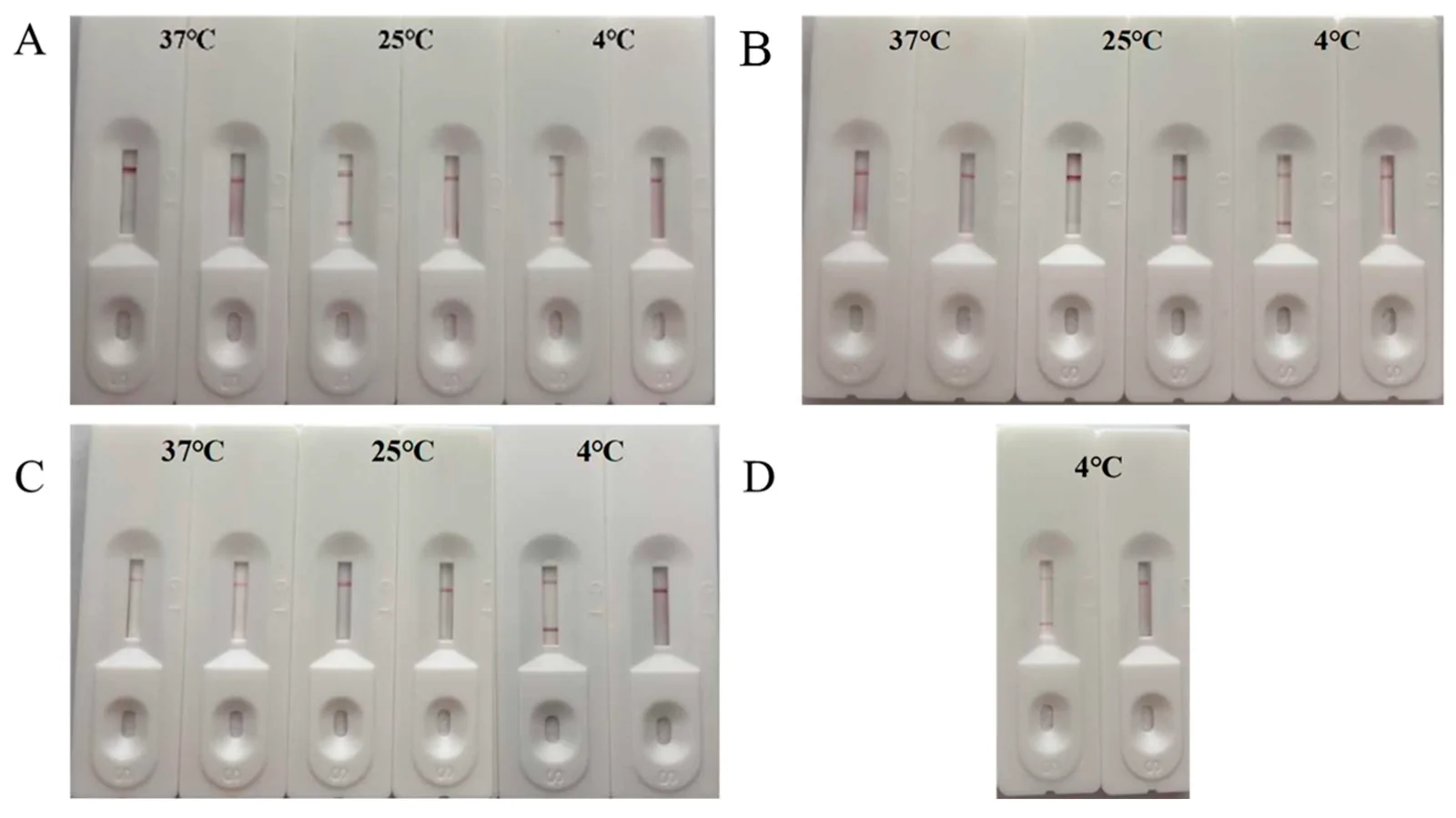
Stability of immunochromatographic strip under different storage conditions: (**A**) 2 months; (**B**) 4 months; (**C**) 6 months; (**D**) 8 months.

The stability and shelf life of the immunochromatographic strip were evaluated under different storage conditions. As shown in Figure 11, after 2 months of storage, strips stored at 37 °C exhibited a faint and unclear T line, whereas those stored at 25 °C and 4 °C still showed clear T lines with good color intensity (Figure 11A). After 4 months, the T-line color intensity of strips stored at 25 °C decreased, while strips stored at 4 °C maintained a clear and distinct T line (Figure 11B). After 6 months, strips stored at 4 °C still showed good T line color development without false negative or false positive results (Figure 11C). Although detectable T-line signals were still observed after 8 months of storage at 4℃ (Fig. 11D), the T-line intensity decreased noticeably compared with that within 6 months. Therefore, considering assay reliability and practical usability, the strip was considered most stable at 4℃, with a conservative shelf life of 6 months.

### 3.9. Detection of Clinical Samples

A total of 200 clinical chicken serum samples were tested simultaneously using the developed immunochromatographic strip and a commercial IBV antibody ELISA kit (IDEXX, USA). The results showed that 84.5% (169/200) and 89.5% (179/200) of the samples were positive according to the strip and ELISA, respectively. The coincidence rate of the two methods was 95.0% (190/200), with a kappa value of 0.78 (*p* < 0.001), indicating a high level of consistency between the two methods (Table 1).

## 4. Discussion

IB brings serious worldwide economic losses to the poultry industry. The IBV genome is prone to mutation and recombination, resulting in increasing variants and poor cross protection between the different serotypes [26,27]. Vaccination is currently an effective measure for preventing and controlling the disease. Rapid and specific antibody detection is the crucial component for conducting disease surveillance, evaluating immunization effectiveness, and implementing epidemic prevention measures. At present, serological methods such as ELISA, IFA, and Western blot are commonly used for the detection of IBV antibodies. However, these methods are relatively time-consuming and generally rely on laboratory infrastructure and trained personnel [28,29,30]. They face difficulty in meeting the rapid and large-scale on-site detection requirements of the poultry industry. Therefore, there is a clear need for a rapid and simple colloidal gold immunochromatographic assay for the detection of IBV antibodies. In the present study, we successfully developed a rapid and simple immunochromatographic strip based on a gold nanoparticle-labeled epitope peptide GST-N2D5 antigen for the detection of IBV antibodies. In our study, the choice of the target is novel and advantageous. The IBV N protein is highly conserved and has strong immunogenicity. It is an advantageous detection target for the early and sustained expression of antibodies. By designing test strips based on conserved epitopes, the assay may reduce the risk of detection failure caused by strain variation and may contribute to broader detection potential, although further validation using samples from different IBV serotypes or variants is still needed. The results can be observed with the naked eye within 5 min, with the most stable results at 16 min, suggesting that the assay may be useful for preliminary on-site detection in poultry farms, pending further field validation. The strip showed high specificity, good sensitivity, and good repeatability. Moreover, the strip demonstrated high agreement with the ELISA. Compared with traditional immunoassays, the colloidal gold immunochromatographic test strip is easy to operate, with rapid detection capabilities and no need for sophisticated equipment. It is suitable for rapid screening of large quantities of samples on site, addressing the pain points of time-consuming laboratory testing, complex operation, and reliance on equipment. To the best of our knowledge, this is the first colloidal gold immunochromatographic strip assay for the detection of IBV antibodies based on a conserved epitope peptide of the N protein.

The N protein is a major structural protein of IBV and has strong immunogenicity and multiple antigenic epitopes [31]. Therefore, it has been widely used as a target antigen in serological assays for IBV. Epitope-based antigens can contribute to assay specificity and diagnostic development [30]. The target antigen used in this study was GST-N2D5, a recombinant epitope-derived protein based on an antigenic epitope of the IBV N protein from the GX-YL5 strain, which represents a dominant genotype and serotype in Guangxi. In our previous work [18], this epitope was shown to react with multiple monospecific sera of IBV, suggesting that it is a suitable antigen candidate for serological detection. At present, immunochromatographic strip methods based on recombinant epitope-derived antigens remain limited. Therefore, the use of the conservative epitope peptide GST-N2D5 in this study provides a useful approach for rapid detection of IBV antibody. This further enriches the rapid serological detection technology for IBV and provides a new and convenient technical means for monitoring the antibody levels of IBV in chicken flocks.

The analytical performance of the GST-N2D5-based strip is particularly important for field application. To evaluate specificity, sera positive for 12 different avian viruses and bacteria were tested. The strip yielded positive results only with IBV-positive sera, indicating that it can distinguish IBV-positive sera from sera positive for other avian pathogens. To assess sensitivity, serial dilutions of IBV-positive sera were tested, and the strip remained positive at a dilution of 1:180, indicating good analytical sensitivity. In addition, the results of the strip showed a high coincidence rate of 95% with those obtained using a commercial IBV antibody ELISA kit, suggesting that the GST-N2D5-based strip can provide rapid and relatively accurate detection of IBV antibodies. The size and homogeneity of AuNPs are critical factors affecting strip performance. Previous studies have shown that larger AuNPs may produce clearer test lines, but excessive particle size can increase steric hindrance and reduce detection sensitivity [32]. AuNPs with a diameter of 40 nm have been reported to provide a favorable balance between signal intensity and analytical performance [33]. In the present study, homogeneous 40 nm AuNPs were prepared by adjusting the concentration of 1% sodium citrate, which may have contributed to the performance of the strip. In addition, assay sensitivity was further improved by optimization of the reaction conditions. GST-tag expression systems are widely used because they facilitate recombinant protein expression and purification [34]. It is generally believed that removal of the GST tag is not always necessary for small epitope-derived proteins. Although GST tag fusion systems facilitate recombinant protein expression and purification, previous studies have shown that fusion tags may influence the diagnostic performance of recombinant antigens in immunoassays, and their effects should therefore be evaluated empirically [35,36].

As mentioned above, the IBV antibody colloidal gold immunochromatographic test strip detection method based on the conserved epitopes of the N protein has the advantages of being rapid, simple, and suitable for preliminary on-site high-throughput screening. However, this test strip also has some drawbacks. Firstly, this strip can only qualitatively detect the presence or absence of antibodies, and cannot distinguish between wild-type infection and vaccine immunity. Secondly, the strip mainly conducts qualitative and semi-quantitative detection, and cannot precisely quantify the antibody titer. Thirdly, due to the limitations of the virus strain variation and the conservation of antigenic determinants, there is a potential risk of missed detection and weak detection. Although this method has the above-mentioned shortcomings, we still believe that it can be used as an effective supplementary technology for precise laboratory detection, forming a “field preliminary screening + laboratory precise examination” complementary detection system.

Further optimizations can be made for the colloidal gold immunochromatographic test strip in the following aspects. Firstly, the detection sensitivity and stability will be further enhanced by selecting more precise conserved epitopes, optimizing antigen coating and reaction systems. Secondly, combined with labeled quantitative technology, achieve precise quantitative detection of antibodies; combine M protein and S protein epitopes to establish a multi-detection system, enabling the differential diagnosis of IBV infection and immunity. Thirdly, although 200 clinical serum samples were used for preliminary validation in this study and the strip showed substantial agreement with a commercial ELISA kit, the sample size and geographical coverage were still limited. In addition, the current study did not include sufficient field samples from different IBV serotypes or variants and different infection or immunization stages. Therefore, further large-scale field studies involving more clinical samples from different regions, different IBV serotypes or variants, and different infection or immunization stages are needed to fully evaluate the diagnostic performance, broad-spectrum applicability, and field application value of the developed strip.

## 5. Conclusions

Overall, a rapid, simple, and practical immunochromatographic strip for IBV antibody detection was successfully established using GST-N2D5, a recombinant epitope-derived antigen based on a conserved B-cell epitope of the IBV N protein. The strip showed good analytical sensitivity and specificity. These findings indicate that the strip has potential as an equipment-free serological tool for field detection and immune monitoring of IBV and may provide new and useful techniques for the prevention and control of IB in poultry industry.

## Figures and Tables

**Table 1:** Comparison of the performance of the immunochromatographic strip and ELISA for detection of anti-IBV antibodies.

Assay		ELISA			Kappa (κ)	*p*-Value of Kappa
		Positive	Negative	Total		
**Test strip**	Positive	169	0	169	0.78	<0.001
	Negative	10	21	31		
	Total	179	21	200		

## Data Availability

The original contributions presented in this study are included in the article. Further inquiries can be directed to the corresponding author.
